# Semiempirical Quantum Chemistry Model for the Lanthanides: RM1 (Recife Model 1) Parameters for Dysprosium, Holmium and Erbium

**DOI:** 10.1371/journal.pone.0086376

**Published:** 2014-01-31

**Authors:** Manoel A. M. Filho, José Diogo L. Dutra, Gerd B. Rocha, Alfredo M. Simas, Ricardo O. Freire

**Affiliations:** 1 Pople Computational Chemistry Laboratory, Departamento de Química, Universidade Federal de Sergipe, São Cristóvão, SE, Brazil; 2 Departamento de Química, CCEN, Universidade Federal da Paraíba, João Pessoa, PB, Brazil; 3 Departamento de Química Fundamental, Universidade Federal de Pernambuco, Recife, PE, Brazil; University of Calgary, Canada

## Abstract

Complexes of dysprosium, holmium, and erbium find many applications as single-molecule magnets, as contrast agents for magnetic resonance imaging, as anti-cancer agents, in optical telecommunications, etc. Therefore, the development of tools that can be proven helpful to complex design is presently an active area of research. In this article, we advance a major improvement to the semiempirical description of lanthanide complexes: the Recife Model 1, RM1, model for the lanthanides, parameterized for the trications of Dy, Ho, and Er. By representing such lanthanide in the RM1 calculation as a three-electron atom with a set of 5 d, 6 s, and 6 p semiempirical orbitals, the accuracy of the previous sparkle models, mainly concentrated on lanthanide-oxygen and lanthanide-nitrogen distances, is extended to other types of bonds in the trication complexes’ coordination polyhedra, such as lanthanide-carbon, lanthanide-chlorine, etc. This is even more important as, for example, lanthanide-carbon atom distances in the coordination polyhedra of the complexes comprise about 30% of all distances for all complexes of Dy, Ho, and Er considered. Our results indicate that the average unsigned mean error for the lanthanide-carbon distances dropped from an average of 0.30 Å, for the sparkle models, to 0.04 Å for the RM1 model for the lanthanides; for a total of 509 such distances for the set of all Dy, Ho, and Er complexes considered. A similar behavior took place for the other distances as well, such as lanthanide-chlorine, lanthanide-bromine, lanthanide, phosphorus and lanthanide-sulfur. Thus, the RM1 model for the lanthanides, being advanced in this article, broadens the range of application of semiempirical models to lanthanide complexes by including comprehensively many other types of bonds not adequately described by the previous models.

## Introduction

Lanthanide complexes, as is well known, have a wide range of high technology applications. Of particular importance is the discovery that, due to their slow magnetization relaxation, lanthanide mononuclear complexes may function as single-molecule magnets [1], [2], the ultimate size limit for spin-based devices. Dysprosium complexes, in particular, will be very important in the development of magnetic materials because of recent results leading to the highest relaxation energy barriers for multinuclear clusters [3], [4], the highest temperature at which hysteresis has been observed for any single complex [5], and a record magnetic blocking temperature of 8.3 K at a sweep rate of 0.08 Ts-1 [6]. Future research, for example, might be directed towards the design of dysprosium complexes that may operate as single-molecule magnets capable of preserving their magnetization at higher and more practical temperatures [6]. Dysprosium complexes are therefore promising for optical storage and memory.

Not only that, both dysprosium and holmium complexes can also effectively function in magnetic resonance imaging, MRI, as negative contrast agents at high magnetic fields, producing darker images, and as agents for susceptibility-induced enhancement at low magnetic fields [7]. Indeed, they are complementary to gadolinium complexes, which act as positive contrast agents, which brighten the image. Indeed, through the simultaneous applications of gadolinium and dysprosium based contrast agents to the MRI diagnosis of conditions such as ischemic heart disease, unprecedented details can now be revealed [8], [9]. Future efforts will likely be intensified towards the design of such MRI contrast agents for the imaging of cellular molecular events involved in normal and pathological processes, including site specific macromolecular and particulate delivery systems [7].

Holmium is also employed in cancer therapeutics due to the characteristics of its ^166^Ho isotope and of its complexes, like 166Ho-DOTMP which has been used in combination with chemotherapy in the treatment of myeloma because it concentrates in metastases of the skeleton and irradiates bone marrow [10].

Erbium (III) luminesces at 1.55 µm, essentially at the center of the third telecommunication window at 1.540 nm. Hence, erbium has been used in long-distance optical transmissions, power amplifiers, repeaters, etc. However, inorganic materials doped with erbium, display a very narrow full width at half maximum [11]. In order to increase the band width, erbium complexes have been used in order to both protect the erbium ion from vibrational coupling, at the same time enhancing the absorption of light through the so-called antenna effect. Indeed, erbium complexes have been prepared that exhibit a much broader full width at half maximum of 68 nm [12], a significant broadening when compared to erbium implanted silica which has a typical value of 11 nm for its most intense peak.

Thus, the design of lanthanide complexes towards enhancement of the property of interest, while seeking to avoid eventual side effects to the health of the subject (where applicable) is an active area of research, which may largely benefit from quantum chemical tools that attempt to predict several of the physical, chemical and even pharmacological [13] properties of the conjectural new structures being considered; structures which might be sketched by assembling around the lanthanide ion, ligands, selected from a library of ligands in a combinatorial manner. And the most important information, from which essentially all quantum chemical property predictions derive, is an accurate geometry of the molecular structure of the complex.

Predictions of geometries of lanthanide complexes from ab initio calculations are not so easy due not only to significant relativistic effects, a consequence of their high atomic numbers, but also to the complex manifold of microstates due, not only to a partially filled f-shell, but also from possibly partially filled 5 d 6 s and 6 p shells [14]. Therefore, full geometry optimizations from such first principles calculations are essentially unfeasible for the technologically useful complexes, which usually exhibit sizes of the order of 100 atoms or more. As a consequence, effective core potentials arise as a practical and very efficient manner of circumventing the complexity, while retaining important characteristics of *ab initio* calculations. Of these, the most widely used are the relativistic pseudopotentials of Dolg [15], [16] which represent an excellent compromise between accuracy and usage of computational resources, mainly computing time. So far, the most thorough study of the geometry prediction accuracy of these relativistic potentials has been carried out by our research group in 2006 when full geometry optimizations were carried out on 52 different lanthanide complexes, including complexes of dysprosium (III), holmium (III) and erbium (III). [17] The counterintuitive results obtained indicated that the best combination of method with basis set when using the MWB pseudopotential was RHF/STO-3G when the intent of the calculation was to predict the geometry of the coordination polyhedron – very important for any subsequent ligand field application. Moreover, either increasing the basis set, or adding electron correlation, only worsened the quality of the resulting coordination polyhedron. On the other hand, although the quality of the obtained coordination polyhedron via RH/STO-3G was very good, that could not be said of the geometry of the attached organic ligands.

In 1994, we introduced the Sparkle Model for the calculation of lanthanide complexes [18], [19], a semiempirical model within the framework of the AM1 semiempirical model [20], which replaced the lanthanide ion by a +3*e* charge, with the corresponding Coulomb field superimposed to a repulsive potential of the form exp(-αr), with α being a parameter designed to somewhat delineate the size of the lanthanide ion, preventing the implosion of the ligands towards it. A very useful method of obtaining absorption spectra of lanthanide complexes was subsequently published [21]. Later [22], Gaussians were added to the core-core repulsion of the sparkle-ligand atom to make the Sparkle Model more consistent with the AM1. In 2005, based on a parameterization scheme employed for europium, gadolinium and terbium [23], the first useful and accurate semiempirical model for dysprosium was defined [24], followed by holmium [25]; and in 2006 for erbium [26]. These models were defined for AM1, and became later available in MOPAC2007 [27], the overall model being called Sparkle/AM1. So far, most applications of the Sparkle Model are related to luminescence research [28]–[30]. But since different semiempirical models possess different accuracies and eventually develop particular niches of applications, it soon became a necessity to extend the Sparkle Model to others, giving rise to Sparkle/PM3 [31], [32], Sparkle/PM6 [33], Sparkle/PM7 [34], targeted to solids, and Sparkle/RM1 [35].

However, none of the above mentioned Sparkle Models attaches semiempirical atomic orbitals to the lanthanide ion. Nevertheless, these models are all very accurate to describe lanthanide-ligand atom distances when the coordinating atom of the ligands is another lanthanide, oxygen or nitrogen. By moving towards other types of lanthanide-ligand atom bonds, however, the accuracy of the Sparkle Models starts to wane. All that points out to the fact that there is some degree of overlap between the orbitals of the lanthanide and those of the coordinating atoms – in short, there is a degree of covalence not taken into account by the Sparkle Model.

In this article, in order to considerably broaden the range of applications of semiempirical methods for lanthanide complexes, we introduce a new model with orbitals for the lanthanide trications of dysprosium, holmium, and erbium, within RM1 [36], which we call simply RM1 model for the lanthanides, a significantly more general model, not to be confused with Sparkle/RM1 [35] which does not have orbitals associated with the lanthanide ion.

## Methods

The rationale of the RM1 model for the lanthanides starts with the following electron configuration for the lanthanide atoms: {[Xe]4f^n^}5d^1^ 6s^2^, with n = 9, 10, 11 for Dy, Ho and Er, respectively. The semiempirical core of the atoms then becomes {[Xe]4f^n^}. The semiempirical valence shells will now have three electrons and will be described by 5 d, 6 s and 6 p orbitals, for a total of 9 orbitals. Hence the model will work for trications only, because for dications there would be a need to parameterize another core of the form {[Xe]4f^n+1^} and assign two electrons to the valence shells, although they could still be described by another set of 5 d, 6 s, and 6 p orbitals. Since trications are by far the most common form of lanthanide ions, as before, we expect the present parameterization to be able to tackle essentially all cases relevant to technological applications.

The next step is to define the universes of complexes, one universe for each of the lanthanide ions under consideration. Accordingly, we selected from the Cambridge Crystallographic Database [37]–[39] all available complexes of high crystallographic quality (R <0.05), for a total of 61 of Dy(III), 40 of Ho(III), and 50 of Er(III).

We then proceeded to select sub-sets of complexes, the parameterization sets, according to some metric capable of guaranteeing that these sub-sets are representative of the universe of complexes with respect to some accuracy measure. Assuming that any difficulties Sparkle/AM1 might be having in describing the coordination polyhedron of the complexes is a reasonable first order approximation to the eventual overall difficulty which the present model will encounter, we defined the following R_i_ metric for each one of the i complexes of the universe for each lanthanide trication:
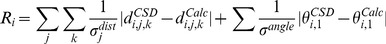
(1)where j runs over all types of bonds, e.g. Ln-N, Ln-O, Ln-C, Ln-S, Ln-P, etc; k, runs over all bonds of type j; 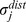
is the standard deviation of all crystallographic bond lengths of type j for all complexes of the universe; 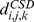 is the crystallographic k^th^ bond distance of type j for complex i; 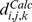
is the calculated value of the same bond; 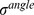 is the standard deviation of all crystallographic bond angles of the type A-Ln-B, with A,B = O, N, C, S, Cl, and Br; 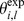 is the crystallographic l^th^ bond angle of complex I; and 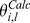 is its calculated counterpart. The standard deviations were calculated from the experimental data only. We also found out that there was no need to split the angles into types, as they all formed a homogeneous set. The divisions of the errors by their corresponding standard deviations make sure that the summations in Eq. (1) add comparable terms. To the set of R_i_ values, each one associated with a different complex, we employed a hierarchical clustering analysis DIANA [40]. DIANA starts out with one large cluster containing all complexes. In the subsequent steps, the complexes that are the most dissimilar are split off into smaller clusters – a procedure which continues until each complex forms a cluster of itself. From the resulting dendogram, we chose two sets of complexes as parameterization sets: a smaller and a larger one. For Dy(III) these sets contained 13 and 26 complexes, respectively. The corresponding numbers for Ho(III) were 12 and 20, and for Er(III) 16, and 39.

The parameterization was carried out to minimize the sum of R_i_s for all complexes of parameterization set, with the difference that the calculated distances and angles in Eq. (1), are now the ones calculated by the model being parameterized. For the parameterization, we used a combination of Simplex and generalized simulated annealing [41] algorithms. We started with the smaller parameterization sets. Once these preliminary optimizations converged, we then expanded the parameterization sets to the larger ones and repeated the process until termination. Table 1 presents the final optimized parameters.

**Table 1:** Parameters* for the RM1 model for the trications of Dy, Ho and Er.

RM1				
Parameter	Unit	Dy^3+^	Ho^3+^	Er^3+^
*U_ss_*	eV	−20.92623973	−22.05745867	−21.97839904
*U_pp_*	eV	−7.66730575	−7.59563761	−7.60784986
*U_dd_*	eV	−17.94081525	−18.00040589	−17.97684107
*ζ_s_*	bohr^−1^	1.29527540	1.33055043	1.34775672
*ζ_p_*	bohr^−1^	1.91210659	1.77955939	1.80648084
*ζ_d_*	bohr^−1^	1.41339670	1.53652417	1.46618905
*β_s_*	eV	−7.60670536	−5.64522644	−5.63471034
*β_p_*	eV	1.96173362	0.00653676	−0.01897203
*β_d_*	eV	−4.36852734	−4.31289917	−4.25067889
*F^0^_sd_*	eV	8.30543139	8.24056943	8.25732681
*G^2^_sd_*	eV	1.31036509	1.24543189	1.24874510
*ρ_core_*	bohr	1.62505501	1.71955962	2.71713627
*α*	Å^−1^	1.34825876	1.33007543	1.32010273
*ζ_s_’*	bohr^−1^	1.37236617	1.49803844	1.44675714
*ζ_p_’*	bohr^−1^	1.07407253	1.96749739	1.97388315
*ζ_d_’*	bohr^−1^	0.81914360	0.66302146	0.65046083
*a_1_*	none	1.13071544	1.09070756	1.17417665
*b_1_*	Å^−2^	7.71195583	7.57151625	7.58325164
*c_1_*	Å	1.53665819	1.49095411	1.50354881
*a_2_*	none	0.06845575	0.00141941	0.00864571
*b_2_*	Å^−2^	7.50653990	7.79969636	7.81378785
*c_2_*	Å	3.23417102	3.25425084	3.23359665

All these parameters are as defined in the formalisms and equations of the RM1 model.

* Parameters are *s*, *p*, and *d* atomic orbital one-electron one-center integrals U_ss_, U_pp_ and U_dd_; the *s*, *p*, and *d* Slater atomic orbital exponents ξ*_s_*, ξ*_p_*, and ξ*_d_*; the *s, p,* and *d* atomic orbital one-electron two-center resonance integral terms *β_s_*, *β_p_*, and *β_d_*; the core-core repulsion term α; the two-electron integrals F^0^_sd_, G^2^_sd_; and the additive term ρ_core_ needed to evaluate core-electron and core-core nuclear interactions; the second set of exponents to compute the one-center integrals *ξ_s_’*, *ξ_p_*’, and *ξ_d_’*; and the six parameters for the two Gaussian functions: height, a_i_; inverse broadness, b_i_; and displacement, c_i_.

## Results and Discussion

In order to evaluate the quality of the optimized parameters, we devised two measures [23], [42]. Both are based on the following formula:
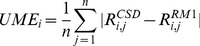
(2)where UME stands for unsigned mean error; i refers to a given complex; n is the number of distances taken into consideration in the given complex; the superscript CSD indicates that the distance R is an experimental crystallographic distance taken from CSD, and the superscript RM1 means that the distance was calculated from the present model. In the first measure we consider only distances between the central lanthanide ion and its directly coordinating atom distances, which we call UME_(Ln-L)._ In the second measure, which we call simply UME, we consider not only the lanthanide ion-directly coordinating atoms as before, but also all distances between all atoms of the coordinating polyhedron, thus indirectly taking into account the angles within the coordination polyhedron.

Tables 2–4 present UME_(Ln-L)_s and UMEs for the universe set of complexes for each of the lanthanide trications: Dy(III), Ho(III), and Er(III), identified by their respective CSD codes.

**Table 2:** Unsigned mean errors, UME_(Dy-L)_s and UMEs, for the RM1 model for the lanthanides, as compared to the respective experimental crystallographic values, obtained from the Cambridge Structural Database, [37]–[39] for each of the 61 dysprosium (III) complexes.

Complex*	RM1		Complex*	RM1	
	UME_(Dy-L)_s (Å)	UME (Å)		UME_(Dy-L)_s (Å)	UME (Å)
AMAQDY	0.0517	0.1503	AKUKAT	0.0216	0.1148
BAFZUE	0.0470	0.1129	BEXLIA	0.0300	0.0972
BIHLIN	0.0317	0.0719	DANPEN	0.0410	0.0953
BUVXIZ01	0.0658	0.0872	DEKBEB	0.0438	0.0832
CECLIF	0.0522	0.0959	DEKBEB01	0.0370	0.0843
CECLIF10	0.0521	0.0960	DEKCAY01	0.0632	0.1068
DIBTIR	0.0313	0.0977	DUFCOW10	0.0348	0.1042
DIDBOH	0.0417	0.1567	FIGXEZ	0.0327	0.1403
FOPNAZ	0.0138	0.1471	FUXPAP01	0.1191	0.1026
FUXRAR	0.0401	0.1662	HIVWEP	0.0665	0.1222
GAKYEW	0.0258	0.1273	HOCYUU	0.0942	0.0976
GINPUO	0.0455	0.0813	IMOXAJ	0.0271	0.1036
HANCAA	0.0553	0.1424	KILZOU02	0.0487	0.1307
KITGEZ	0.0480	0.1391	KUYBIP	0.1338	0.1260
LEYHUS	0.0296	0.2200	LEZZOG	0.0343	0.0741
MANHOY	0.0804	0.1208	MECCUT	0.0408	0.1836
PALBIN	0.0205	0.4919	NAKMAO	0.0323	0.0713
QQQEMM01	0.0328	0.0841	NAPHAN	0.0377	0.0883
SETADY	0.0883	0.2516	OHUYUM	0.0286	0.0985
TISQUH	0.0670	0.1240	RABBEX	0.0825	0.2034
TUQTUU	0.0601	0.0940	ROCTIN	0.1118	0.2276
TUQTUU01	0.0601	0.0943	TESHEF	0.0348	0.1448
VOSBOU	0.0587	0.0879	TESHOP	0.0571	0.1010
XAWVIA	0.0408	0.1087	TESJEH	0.0356	0.1150
XEQMAH	0.0987	0.1019	TESJIL	0.0564	0.1008
XIVFUD	0.1015	0.2004	USEPEO	0.0484	0.1090
YAVSOD	0.0432	0.0726	WAQZEU	0.1499	0.1742
ZAXSAS	0.0700	0.1357	WAWJOT01	0.1152	0.1538
ZZZARG01	0.0602	0.0897	WEDHUJ	0.0282	0.1089
AGUTOL	0.0719	0.1019	XAYRIZ	0.0416	0.0874
AHANED	0.0817	0.1433			

* The complexes are identified by their unique CSD codes [37]–[39].

**Table 3:** Unsigned mean errors, UME_(Ho-L)_s and UMEs, for the RM1 model for the lanthanides, as compared to the respective experimental crystallographic values, obtained from the Cambridge Structural Database, [37]–[39] for each of the 40 holmium (III) complexes.

Complex*	Method RM1		Complex*	Method RM1	
	UME_(Ho-L)_s (Å)	UME (Å)		UME_(Ho-L)_s (Å)	UME (Å)
BAGBAN	0.0513	0.1131	XARVOB	0.0363	0.0912
BEYSAZ	0.0529	0.1976	XAWVOG	0.0530	0.0930
BUVXOF01	0.0634	0.0869	XEQMEL	0.0843	0.0602
CAQFUV	0.0676	0.0790	XEWVIE	0.0696	0.1305
COZHEE	0.0223	0.0966	XORGEQ	0.0362	0.4482
CUSYUK	0.0483	0.2318	AGUVED	0.0443	0.0838
ECOJEL	0.0975	0.1573	AXAZAA	0.0893	0.1137
FAGYOC	0.0695	0.3482	DEKBAX	0.0378	0.0768
GAKYIA	0.0462	0.1312	DEKBOL	0.1163	0.1557
GINREA	0.0460	0.0775	EWIPUV	0.0365	0.1275
GODKOZ	0.0358	0.0523	FUXRIZ	0.0742	0.0715
HANCII	0.0196	0.0593	GIFLIQ	0.1142	0.1489
HOESUL02	0.0621	0.0916	MOGWUB	0.4893	0.6047
KITGOJ	0.0254	0.0773	MUHWIW	0.0491	0.0888
LEYJEE	0.0413	0.1874	NUFQAG	0.0399	0.0715
LIZPAL	0.0499	0.2272	NUYNOL	0.0689	0.0652
NIHRIF	0.0640	0.1070	NUYNOL01	0.0484	0.0764
NUJBAV	0.0087	0.0625	QELLOJ	0.0493	0.0937
QOZVOQ	0.0185	0.1002	QIVYAW	0.0423	0.0620
SIFZIQ	0.0273	0.1721	YEFVUA	0.0349	0.0760

* The complexes are identified by their unique CSD codes [37]–[39].

**Table 4:** Unsigned mean errors, UME_(Er-L)_s and UMEs, for Method RM1, as compared to the respective experimental crystallographic values, obtained from the Cambridge Structural Database, [37]–[39] for each of the 59 erbium (III) complexes.

Complex*	Method RM1		Complex*	Method RM1	
	UME_(Er-L)_s(Å)	UME(Å)		UME_(Er-L)_s(Å)	UME(Å)
AERETS02	0.0563	0.0895	RIKTEK	0.0522	0.2208
AKIYEY	0.0440	0.2700	RIKTEK01	0.0488	0.2197
BAGBER	0.0462	0.1076	ROCSOS	0.0782	0.2444
BEXLEW	0.0842	0.1678	ROCTOT	0.0595	0.2138
BOBWAQ	0.0421	0.1184	RUNQOG	0.0513	0.1022
BODMUD	0.0388	0.1382	SEGVAB	0.0691	0.1681
BOWXOA	0.0450	0.1848	SOKBID	0.0280	0.0513
DEKCEC	0.0371	0.0914	TACERB01	0.0262	0.0958
DIBTAJ	0.0369	0.1778	TEJFEU	0.1355	0.2048
DIDCAU	0.0399	0.2601	TEPKOO	0.0297	0.0715
DIJQAO	0.0391	0.2332	TMHDER	0.0588	0.3224
DIJQIW	0.0310	0.1024	TUMJEQ	0.0583	0.1227
DIYNII	0.0260	0.1158	UFIRIK	0.0622	0.0716
DOGKEP	0.0323	0.1502	VEQFOM	0.0820	0.0617
GAKYOG	0.0266	0.1057	VOSNOG	0.0375	0.0833
GINRIE	0.0464	0.0807	VUSGUL	0.0817	0.0941
HANCOO	0.0165	0.0609	VUSHEW	0.0593	0.1107
HEDVIW	0.1052	0.1989	WEFVIM	0.0466	0.0843
HENAEB	0.0283	0.1548	XAXYAX	0.0593	0.0854
KITGUP	0.0149	0.0729	XEWVOK	0.0442	0.1163
KOZBUW	0.0144	0.0561	XEWWUR	0.0472	0.0938
LEYJII	0.0294	0.2276	XOVHAS	0.0851	0.2268
MAGDOP	0.0449	0.1319	XOYXIS	0.0285	0.1590
MECDEE	0.0281	0.0853	YEGFEV	0.0504	0.1687
NIVQUE	0.0499	0.2188	YEMSIT	0.0767	0.1338
NUYNUR	0.0838	0.2163	YICCIW	0.0344	0.1527
OHUZEX	0.0269	0.0890	YUFWIG	0.0196	0.0988
OMATUS	0.0742	0.1597	ZADWUW	0.1281	0.2265
QIVXID	0.0742	0.2170	ZUFSAU	0.0480	0.1304
RELNIG	0.0530	0.1011			

* The complexes are identified by their unique CSD codes [37]–[39].

We now proceed to the statistical validation of the model [43]. If the parameterizations captured the essence of the coordinating bonds, then the histograms of both UME_(Ln-L)_ and UME must follow gamma distribution functions since, by definition, the UMEs can only have positive values. The gamma distributions are then adjusted to reproduce the mean and variance of the UME_(Ln-L)_s, for each of the parameterized trications. Finally, the qualities of the gamma distribution fits of the data were then assessed via the one-sample nonparametric Kolmogorov-Smirnoff test [44]. If the p-value of the test is larger than 0.05, then the gamma distribution fit is justified within a 95% interval and the use of the mean and variance of the data, as accuracy measures, is also statistically justified. Accordingly, Tables 5 and 6 display the mean, variance, and p-value of the test for each of the lanthanide ions, for both the UME_(Ln-L)_s and UMEs. All p-values are substantially larger than 0.05 and, therefore, the means and variances in Tables 5 and 6 can justifiably be taken as accuracy measures of the models for Dy(III), Ho(III), and Er(III).

**Table 5:** Means and variances for the *γ* distribution fits of the UME_(Ln-L)_s computed for the *N* complexes for each lanthanide trication.

UME_(Ln-L)_s				
Lanthanide ion	N	mean (Å)	variance (Å^2^)	p-value
Dy^3+^	61	0.0539	0.0032	0.7986
Ho^3+^	40	0.0602	0.0069	0.1292
Er^3+^	58	0.0506	0.0025	0.9082

**Table 6:** Means and variances for the *γ* distribution fits for the UMEs computed for the *N* complexes for each lanthanide trication.

UMEs				
Lanthanide ion	N	mean (Å)	variance (Å^2^)	p-value
Dy^3+^	61	0.1193	0.0169	0.1578
Ho^3+^	40	0.1225	0.0290	0.1463
Er^3+^	58	0.1348	0.0228	0.5425

**Figure 1:**
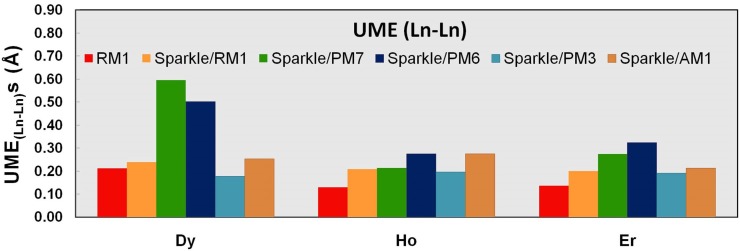
UME_(Ln-Ln)_s obtained using the RM1 model for the lanthanides and all five versions of the Sparkle Model: Sparkle/RM1, Sparkle/PM7, Sparkle/PM6, Sparkle/PM3 and Sparkle/AM1 for all complexes of the universe set for each of the lanthanide trications: Dy(III), Ho(III) and Er(III). The UMEs are calculated as the absolute value of the difference between the experimental and calculated Ln-Ln interatomic distances, summed up for all complexes, for each of the lanthanides.

**Figure 2:**
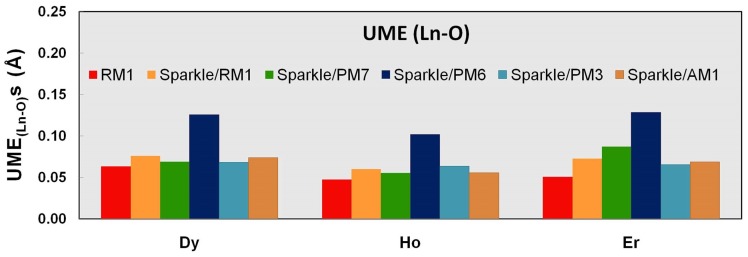
UME_(Ln-O)_s obtained using the RM1 model for the lanthanides and all five versions of the Sparkle Model: Sparkle/RM1, Sparkle/PM7, Sparkle/PM6, Sparkle/PM3 and Sparkle/AM1 for all complexes of the universe set for each of the lanthanide trications: Dy(III), Ho(III) and Er(III). The UMEs are calculated as the absolute value of the difference between the experimental and calculated Ln-O interatomic distances, summed up for all complexes, for each of the lanthanides.

**Figure 3:**
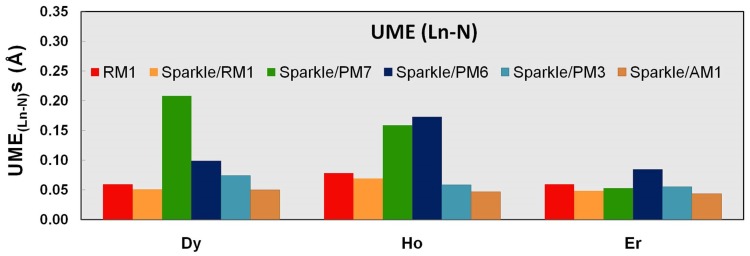
UME_(Ln-N)_s obtained using the RM1 model for the lanthanides and all five versions of the Sparkle Model: Sparkle/RM1, Sparkle/PM7, Sparkle/PM6, Sparkle/PM3 and Sparkle/AM1 for all complexes of the universe set for each of the lanthanide trications: Dy(III), Ho(III) and Er(III). The UMEs are calculated as the absolute value of the difference between the experimental and calculated Ln-N interatomic distances, summed up for all complexes, for each of the lanthanides.

We now proceed to analyze the performance of the models with respect to specific types of distances for Dy(III), Ho(III), and Er(III). Tables 7–9 and Figures 1–3 show UMEs for all types of Ln-L distances present in the universe of Ln(III) complexes, together with the corresponding values from the previous sparkle models for comparison. It must be noted, though, that in the original sparkle model articles, we only included complexes with exclusively Ln-O and Ln-N bonds. But, in the present article, we are considering a much larger set of complexes with other types of bonds such as Ln-C, Ln-Cl, etc. Indeed, here we now may have complexes which have not only Ln-O and/or Ln-N bonds, but also other types of bonds, such as Ln-C bonds, all in the same compound. Of course, these were not included as test cases for the previous Sparkle models, but are here taken in full consideration. And that is the reason why UMEs for the Ln-O and Ln-N types of bonds in the present article tend to be different, slightly larger, when set side by side to similar Ln-O and Ln-N UMEs of the original sparkle model articles. However, not to unnecessarily crowd the present article, in the tables, we only show numbers computed using the old models, but for the new test set.

**Table 7:** RM1, Sparkle/RM1, Sparkle/PM7, Sparkle/PM6, Sparkle/PM3, and Sparkle/AM1 unsigned mean errors for different types of distances of dysprosium(III) complexes.

Type of distances	unsigned mean errors for specific types of distances (Å)						
	N	RM1	Sparkle/RM1	Sparkle/PM7	Sparkle/PM6	Sparkle/PM3	Sparkle/AM1
Dy - Dy	16	0.2118	0.2397	0.5950	0.5025	**0.1784**	0.2531
Dy - O	283	**0.0637**	0.0760	0.0692	0.1259	0.0685	0.0740
Dy - N	105	0.0594	0.0510	0.2084	0.0992	0.0741	**0.0500**
Dy - C	315	**0.0341**	0.1854	0.2567	0.4616	0.2346	0.2161
Dy - S	21	**0.0824**	0.5016	0.9833	0.5359	0.4996	0.5019
Dy - P	3	**0.0273**	0.1591	2.3194	0.3934	0.4205	0.3918
Dy - Cl	20	**0.0525**	0.2858	0.1757	0.2318	0.2475	0.2546
Dy - Br	5	**0.0311**	0.4564	1.3418	0.3986	0.4209	0.4320
Dy - L	768	**0.0539**	0.1408	0.2209	0.2846	0.1598	0.1527
L-L′	3499	**0.1336**	0.2370	0.2931	0.3449	0.2433	0.2621
Dy-L, Dy-Dy and L-L′	4267	**0.1193**	0.2197	0.2801	0.3341	0.2283	0.2424

**Table 8:** RM1, Sparkle/RM1, Sparkle/PM7, Sparkle/PM6, Sparkle/PM3, and Sparkle/AM1 unsigned mean errors for specific types of distances for holmium(III) complexes.

Type of distances	unsigned mean errors for specific types of distances (Å)						
	N	RM1	Sparkle/RM1	Sparkle/PM7	Sparkle/PM6	Sparkle/PM3	Sparkle/AM1
Ho - Ho	4	**0.1301**	0.2083	0.2144	0.2751	0.1958	0.2747
Ho - O	219	**0.0475**	0.0604	0.0554	0.1021	0.0639	0.0557
Ho - N	58	0.0786	0.0696	0.1592	0.1732	0.0585	0.**0469**
Ho - C	98	**0.0752**	0.2256	0.4544	0.5380	0.2537	0.2655
Ho - Cl	28	**0.0585**	0.3055	0.1262	0.2777	0.2480	0.2679
Ho - L	407	**0.0602**	0.1198	0.1727	0.2310	0.1228	0.1217
L – L′	1748	**0.1371**	0.2326	0.3289	0.3462	0.2324	0.2585
Ho-L, Ho-Ho and L-L	2155	**0.1225**	0.2113	0.2994	0.3245	0.2117	0.2327

**Table 9:** RM1, Sparkle/RM1, Sparkle/PM7, Sparkle/PM6, Sparkle/PM3, and Sparkle/AM1 unsigned mean errors for specific types of distances for erbium complexes.

Type of distances	unsigned mean errors for specific types of distances (Å)						
	N	RM1	Sparkle/RM1	Sparkle/PM7	Sparkle/PM6	Sparkle/PM3	Sparkle/AM1
Er - Er	6	**0.1200**	0.2639	0.5458	0.2124	0.2439	0.2626
Er - O	336	**0.0509**	0.0730	0.0874	0.1285	0.0657	0.0689
Er - N	77	0.0594	0.0484	0.0529	0.0846	0.0551	**0.0434**
Er - C	96	**0.0318**	0.2004	0.4280	0.5474	0.2277	0.2177
Er - S	12	**0.1088**	0.4802	1.2501	0.5234	0.5211	0.5212
Er - Cl	33	**0.0574**	0.3243	0.3406	0.2753	0.2975	0.2928
Er - Br	3	**0.0463**	0.4526	1.5964	0.4475	0.4146	0.4291
Er - L	563	**0.0511**	0.1199	0.1967	0.2136	0.1205	0.1189
L – L′	2259	**0.1575**	0.2197	0.2938	0.3519	0.2076	0.2371
Er-L, Er-Er and L-L′	2822	**0.1363**	0.2000	0.2746	0.3243	0.1904	0.2137

Dy-Dy distances in dilanthanide complexes of Dy (III), Ho (III), and Er (III) lie in the range from 3.6 Å to 6.6 Å, while lanthanide-other ligand atom distances lie on average around 2.5 Å. That is why Ln-Ln UMEs are larger than other Ln-L UMEs. The previous Sparkle Models focused on these Ln-Ln, a also on Ln-O, and Ln-N distances only. Indeed, considering only Dy complexes (Table 7), there are 404 distances of these types, which represent 53% of all distances involving Dy(III) in its universe of complexes. The next most important types are Dy(III)-C distances, for which there are 315 of them making up 41% of the total.

**Figure 4:**
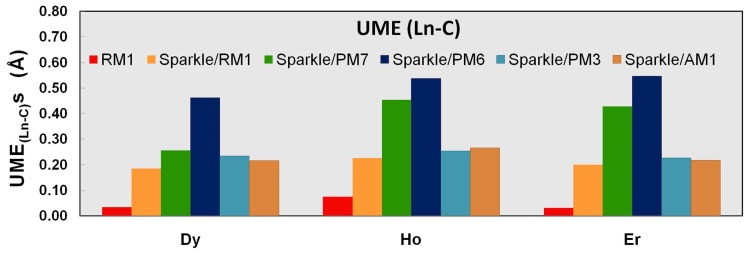
UME_(Ln-C)_ obtained using the RM1 model for the lanthanides and all five versions of the Sparkle Model: Sparkle/RM1, Sparkle/PM7, Sparkle/PM6, Sparkle/PM3 and Sparkle/AM1 for all complexes of the universe set for each of the lanthanide trications: Dy(III), Ho(III) and Er(III). The UMEs are calculated as the absolute value of the difference between the experimental and calculated Ln-C interatomic distances, summed up for all complexes, for each of the lanthanides.

By examining Table 7 and Figure 4, we can see a significant improvement in these next types of distances, with UME_ (Dy-C)_s for the RM1 model for the lanthanides being 0.03 Å, whereas the corresponding average value of the sparkle models is 0.27 Å, a value 9 times larger. That alone would justify the introduction of the RM1 model for the lanthanides because, in the case of dysprosium, almost half the extant Ln-L distances are significantly more accurately described by RM1.

**Figure 5:**
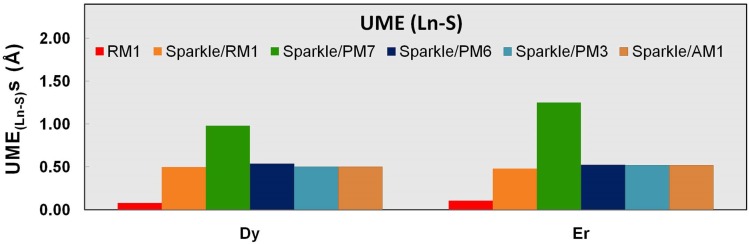
UME_(Ln-S)_ obtained using the RM1 model for the lanthanides and all five versions of the Sparkle Model: Sparkle/RM1, Sparkle/PM7, Sparkle/PM6, Sparkle/PM3 and Sparkle/AM1 for all complexes of the universe set for each of the lanthanide trications: Dy(III), Ho(III) and Er(III). The UMEs are calculated as the absolute value of the difference between the experimental and calculated Ln-S interatomic distances, summed up for all complexes, for each of the lanthanides. There are no Ho-S distances in the universe of Ho(III) complexes considered.

**Figure 6:**
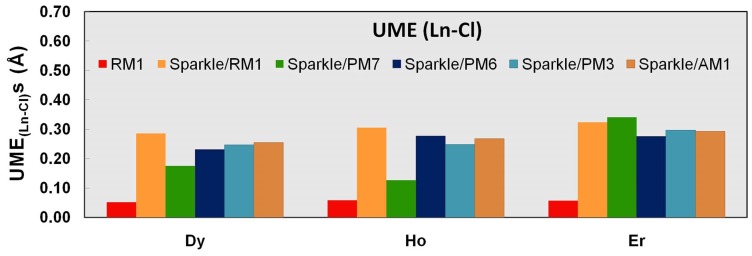
UME_(Ln-Cl)_s obtained using the RM1 model for the lanthanides and all five versions of the Sparkle Model: Sparkle/RM1, Sparkle/PM7, Sparkle/PM6, Sparkle/PM3 and Sparkle/AM1 for all complexes of the universe set for each of the lanthanide trications: Dy(III), Ho(III) and Er(III). The UMEs are calculated as the absolute value of the difference between the experimental and calculated Ln-Cl interatomic distances, summed up for all complexes, for each of the lanthanides.

**Figure 7:**
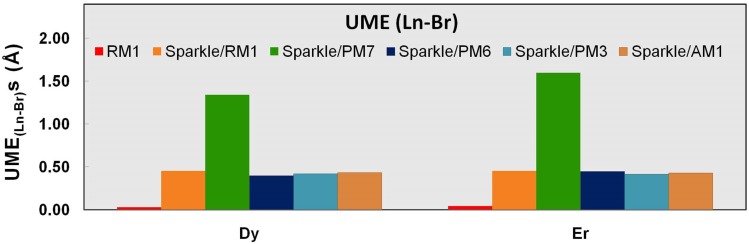
UME_(Ln-Br)_s obtained using the RM1 model for the lanthanides and all five versions of the Sparkle Model: Sparkle/RM1, Sparkle/PM7, Sparkle/PM6, Sparkle/PM3 and Sparkle/AM1 for all complexes of the universe set for each of the lanthanide trications: Dy(III), Ho(III) and Er(III). The UMEs are calculated as the absolute value of the difference between the experimental and calculated Ln-Br interatomic distances, summed up for all complexes, for each of the lanthanides. The RM1 model for lanthanides UME_(Ln-Br)_ bars are very small. Besides, there are no Ho-Br distances in the universe of Ho(III) complexes considered.

The situation is less dramatic but still significant for the other trications being parameterized, when Ln-C distances represent 24% of the total for Ho(III), and 17% for Er(III). The RM1 model for the lanthanides is even further justified when we compare other types of less common distances like Ln-S, Ln-Cl, and Ln-Br, because it outperforms all previous sparkle models as shown in Tables 7–9 and Figures 5–7. In all these instances, the RM1 UMEs tend to be almost ten times smaller than the corresponding errors of all previous sparkle models.

**Figure 8:**
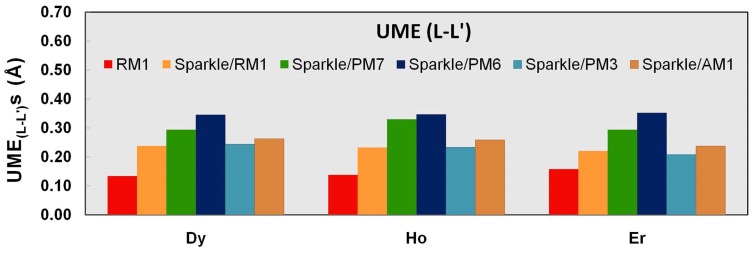
UME_(L-L′)_s obtained using the RM1 model for the lanthanides and all five versions of the Sparkle Model: Sparkle/RM1, Sparkle/PM7, Sparkle/PM6, Sparkle/PM3 and Sparkle/AM1 for all complexes of the universe set for each of the lanthanide trications: Dy(III), Ho(III) and Er(III). The UMEs are calculated as the absolute value of the difference between the experimental and calculated interatomic distances between the coordinated atoms, L-L′, summed up for all complexes, for each of the lanthanides.

Finally, we can have an idea of the accuracy of the angles by examining the distances between any two atoms of the coordination polyhedron, the L-L′distances. For all three lanthanide trications, there was a significant reduction of these UMEs by a factor of two when compared to the corresponding UMEs for the previous sparkle models: from 0.26 Å to 0.13 Å, as can be inferred from Figure 8. This is indirect evidence that the angles are much better predicted in the RM1 model for the lanthanides.

## Conclusions

The RM1 model for the lanthanides represents a significant improvement in the theoretical semiempirical modeling of lanthanide complexes, which started with the sparkle model in 1994 [18], [19] which and attained maturity with the introduction of Sparkle/AM1 in 2005 [23], and was extended to Sparkle/PM3 [31], [32], Sparkle/PM6 [33], to Sparkle/PM7 [34] and up to Sparkle/RM1 [35], the last two in 2013.

There is, however, a cost associated with the improvement. The RM1 model for the lanthanides adds nine more orbitals per lanthanide to the calculation, whereas all sparkle models add none. Thus, for a single SCF calculation of a complex of about 60 atoms, for example, an RM1 model for the lanthanides calculation takes about twice the computing time of a Sparkle/RM1 calculation. Such cost can become even weightier if the molecular structure contains more than one lanthanide ion, as is usually the case of metal-organic frameworks. Since the performance of both Sparkle/RM1 and RM1 model for the lanthanides is essentially equivalent for Ln-Ln, Ln-O, and Ln-N, the user may still benefit from the speed of the sparkle models if the structure of interest contains only these types of bonds, as it takes place in the majority of cases.

In conclusion, via the introduction of a set of 5 d, 6 s, and 6 p semiempirical atomic orbitals, the RM1 model for the lanthanides thus extends the Sparkle Models’ capabilities of correctly describing Ln-Ln, Ln-O, and Ln-N distances, to other types of distances, such as Ln-C, Ln-S, Ln-P, Ln-Cl, and Ln-Br, while simultaneously improving the coordinating bond angles.

## Supporting Information

File S1 **Instructions on how to run the RM1 model for the lanthanides in MOPAC12** [**45**] **, and MOPAC sample input and output files for complexes of Dy(III), Ho(III), and Er(III).**
(DOC)
