# Supplementary material for: Semiempirical Quantum Chemistry Model for the Lanthanides: RM1 (Recife Model 1) Parameters for Dysprosium, Holmium and Erbium
Source: PLoS One. 2014 Jan 31;9(1):e86376. doi: 10.1371/journal.pone.0086376 (PMC3908927; doi:10.1371/journal.pone.0086376)
Supplement: File S1 — Instructions on how to run the RM1 model for the lanthanides in MOPAC12 [45] , and MOPAC sample input and output files for complexes of Dy(III), Ho(III), and Er(III). (DOC) [file pone.0086376.s001.doc]

Semiempirical Quantum Chemistry Model for the Lanthanides: RM1 (Recife Model 1) Parameters for Dysprosium, Holmium and Erbium

Supporting Information

Manoel A. M. Filho^1^, José Diogo L. Dutra^1^, Gerd B. Rocha^2^, Alfredo M. Simas^3^, and Ricardo O. Freire^1*^

^1^Pople Computational Chemistry Laboratory, Departamento de Química, Universidade Federal de Sergipe, 49.100-000 – São Cristóvão, SE, Brazil.

^2^Departamento de Química, CCEN, Universidade Federal da Paraíba, 58.059-970 – João Pessoa, PB, Brazil.

^3^Departamento de Química Fundamental, Universidade Federal de Pernambuco, 50.740-540, Recife, PE, Brazil.

^*^Corresponding author. E-mail: [rfreire@ufs.br](mailto:rfreire@ufs.br)

Contents

| 1. [How to run RM1 model for the lanthanides calculations with MOPAC2012.](#link13) ………………. | 2 |
| --- | --- |
| 1. [MOPAC2012 Input (.mop) and output (.arc) files.](#link3) ……………………………………………….…….. | 4 |
| 1. [Graphical User Interfaces for MOPAC2012](#link4) ………………………………………………..………………. | 4 |
| 1. [MOPAC Sample Input and Output Files](#link6)………………………………………………………….…………… | 5 |
| 1. [References](#link6)…………………………………………………………………………………………..……………………… | 16 |

1. **How to run RM1 model for the lanthanides calculations with MOPAC2012**

([back to contents](#Contents))

MOPAC2012 is the new software released by Prof. James J. P. Stewart from *Stewart Computational Chemistry* of Colorado Springs, CO, and represents the most recent version of the MOPAC series of molecular modeling softwares, which started in 1981.

MOPAC2012 has Sparkle/AM1, Sparkle/PM3, Sparkle/PM6, Sparkle/PM7, and Sparkle/RM1 fully implemented. Instructions on how to use the Sparkle Model in MOPAC2012, and on how to visualize the complexes with graphical user interfaces, can be found at [http://www.sparkle.pro.br](http://www.sparkle.pro.br/).

A MOPAC2012 executable can be obtained from [http://openmopac.net](http://openmopac.net/) and is presently free for academics.

In order to be acquainted with the software, users are encouraged to read the MOPAC2012 manual at <http://openmopac.net/manual/>.

As the MOPAC2012 manual says:

*MOPAC is written with the non-theoretician in mind.*

*While MOPAC calls upon many concepts in quantum theory and thermodynamics and uses some fairly advanced mathematics, the users need not be familiar with these specialized topics.*

At present, the most recent version of MOPAC2012 is 12.236W.

To run a RM1 model for lanthanides calculation in MOPAC 2012, proceed as follows:

1. Create a data-file with extension .mop which describes a molecular system and specifies the type of calculation that is to be carried out.
   1. Use **only** the keyword [RM1](http://openmopac.net/manual/rm1_key.html). Do not forget to set the charge n of the complex with keyword [CHARGE=n](http://openmopac.net/manual/charge.html)

To run a Sparkle/RM1 calculation in MOPAC 2012, proceed as follows:

- 1. Use the lanthanide as you would use any atom in MOPAC.
  2. Do not forget to specify the charge n of the complex with keyword CHARGE=n
  3. For a Sparkle/RM1 calculation, use the keywords [RM1](http://openmopac.net/manual/am1.html) [SPARKLE](http://openmopac.net/manual/sparkle.html) in the keyword line.

1. Command MOPAC to run the calculation using that data-file.
2. Get the desired output on the system from the output files created by MOPAC.
3. **MOPAC2012 Input (.mop) and output (.arc) files** ([back to contents](#Contents))

Sample input and output files for all Sparkle Models can be found in [http://www.sparkle.pro.br](http://www.sparkle.pro.br/).

As examples, we are providing in the appendix of this supplementary material the content of a MOPAC2012 input and the corresponding RM1 output file for one complex for each lanthanide ion.

In order to reproduce the calculation, please [request a password and download](../../../../E:%5CPapers_Colaborações%5CSimas_SparklePM7%5CPaper_Sparkle_PM7%5Crequest%20a%20password%20and%20download) MOPAC2012.exe from [http://openmopac.net](http://openmopac.net/), which is presently free for academics. Then, copy the contents of one of the sample inputs to a text file, name it something like sample.mop, and simply open it with MOPAC2012.

Warning: MOPAC2012 output files with extension .arc may be confused with some types of compressed files in some Windows systems. Be sure to open them with notepad, or a similar text editor.

*At present, this supplementary material shows output files from a version of MOPAC2007 we used to implement the RM1 model for lanthanides. As soon as the paper is accepted, this model will be implemented in MOPAC2012 and this supplementary information will be updated accordingly.*

1. **Graphical User Interfaces for MOPAC2012** ([back to contents](#Contents))

A large number of graphical user interfaces, GUIs, that can be used with MOPAC2012, both commercial and free, can be found [here](http://openmopac.net/resellers.html).

Warning: the bond connection algorithm of some of the Graphical User Interfaces may not work efficiently with some high coordination number lanthanide complexes. Some coordinating bonds may not appear, while sometimes some other spurious bond connections may also appear. However, the positions of the atoms are always correct.

1. **MOPAC Sample Input and Output Files** ([back to contents](#Contents))

The complexes are identified by their Cambridge Structural Database unique structure codes^1-3^.

**Dysprosium: YAVSOD
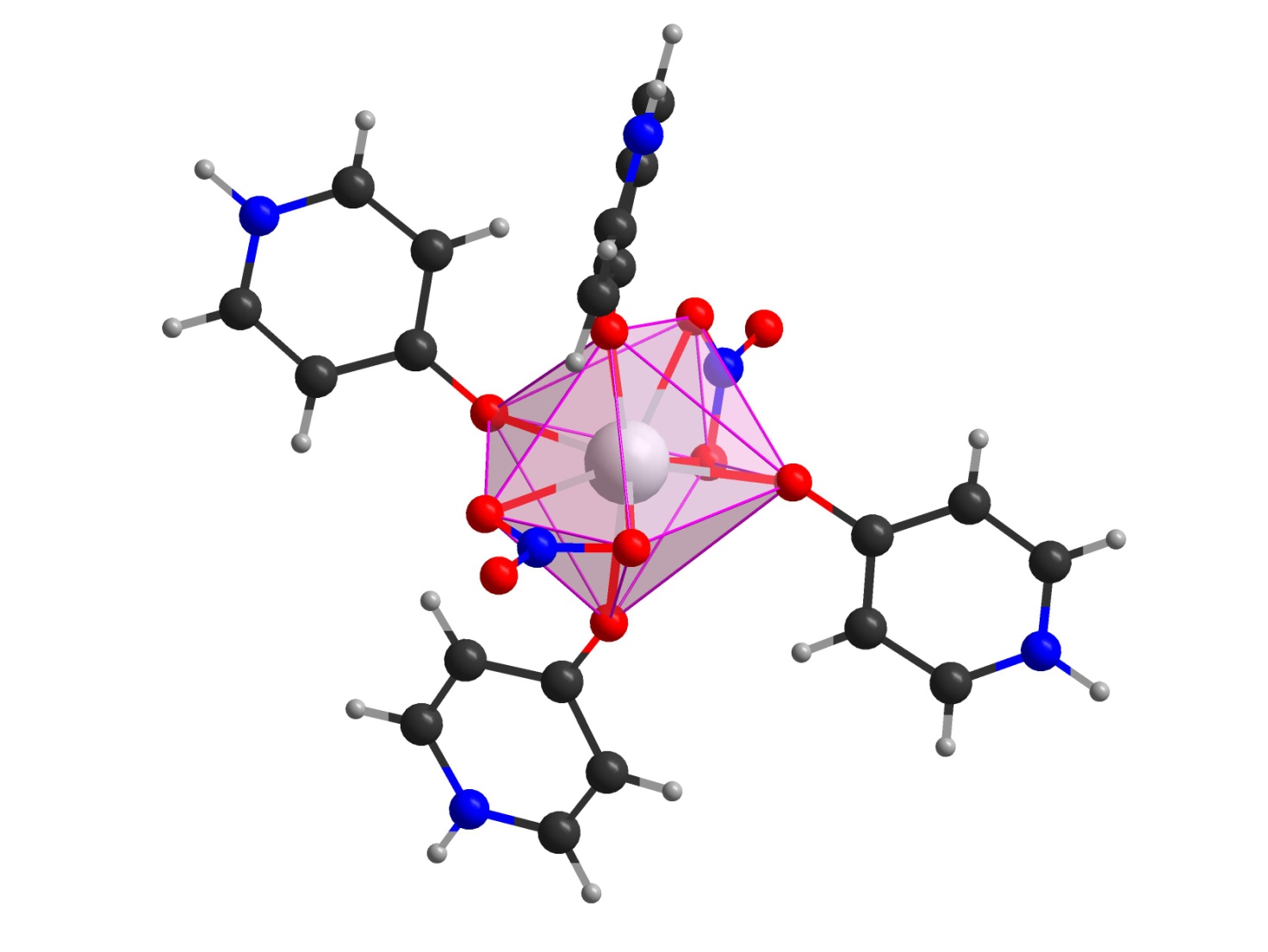
**

---------------------------------------- Begin of file **YAVSOD.mop**-----------------------------------

RM1 EXTERNAL=spk.inp PRECISE NOINTER XYZ BFGS T=10D GNORM=0.25 +

NOLOG GEO-OK SCFCRT=1.D-10 CHARGE=1.0

NUMERO DE COORDENAÇÃO= 8

Dy 0.0000000 1 0.0000000 1 0.0000000 1

O 0.6955041 1 -2.1153184 1 0.3596589 1

O 2.0883539 1 0.8937307 1 0.2618099 1

O -1.8821924 1 -0.7164727 1 -0.9937568 1

O -0.9904109 1 1.9368201 1 0.4963498 1

O 1.0695894 1 -0.6100318 1 -2.1122697 1

O 0.0687844 1 1.2849361 1 -2.1248946 1

O 0.4334275 1 0.1567564 1 2.4575742 1

O -1.4843513 1 -0.5947143 1 1.8863743 1

O 1.0498359 1 0.5965601 1 -3.9226333 1

O -1.0607586 1 -0.2499103 1 3.9616668 1

N -0.7106468 1 -0.2363340 1 2.8128071 1

N 0.7347188 1 0.4243194 1 -2.7628604 1

N -1.7856878 1 5.6029678 1 1.9639058 1

N -5.7569984 1 0.4193065 1 -1.2235407 1

N 0.1458031 1 -6.0085821 1 -0.5437841 1

N 6.0349000 1 0.0000000 1 0.0000000 1

C 5.6313562 1 1.2708690 1 0.0000000 1

C 4.3262363 1 1.5940769 1 0.0506851 1

C 3.3495613 1 0.5994756 1 0.1442938 1

C 3.8052868 1 -0.7285603 1 0.0984914 1

C 5.1431868 1 -0.9782544 1 0.0404545 1

C 1.3357946 1 -5.5803110 1 -0.1947167 1

C 1.5461717 1 -4.2890124 1 0.1303420 1

C 0.4875007 1 -3.3538896 1 0.0901850 1

C -0.7732820 1 -3.8486797 1 -0.2614543 1

C -0.9148883 1 -5.1650466 1 -0.5798981 1

C -5.4550237 1 -0.8682943 1 -1.0415508 1

C -4.1619902 1 -1.2773170 1 -0.9605632 1

C -3.1205470 1 -0.3556712 1 -1.0700621 1

C -3.4899003 1 0.9842962 1 -1.2935937 1

C -4.7943182 1 1.3332401 1 -1.3421221 1

C -1.7892245 1 5.4097845 1 0.6529681 1

C -1.5966393 1 4.1724102 1 0.1291834 1

C -1.3160930 1 3.0846118 1 0.9543776 1

C -1.4115670 1 3.3321559 1 2.3399032 1

C -1.6142416 1 4.5892322 1 2.8017709 1

H 6.9715152 1 -0.2057690 1 -0.0293152 1

H 6.2877985 1 1.9706330 1 -0.0373894 1

H 4.0635981 1 2.5161895 1 0.0225032 1

H 3.1781293 1 -1.4557005 1 0.1092958 1

H 5.4478948 1 -1.8872455 1 0.0268277 1

H 0.0242940 1 -6.9323533 1 -0.7767013 1

H 2.0707771 1 -6.1971536 1 -0.1718668 1

H 2.4227852 1 -4.0026157 1 0.3934134 1

H -1.5349075 1 -3.2651403 1 -0.2769624 1

H -1.7770159 1 -5.5002098 1 -0.8330436 1

H -6.6774059 1 0.6890371 1 -1.2691410 1

H -6.1620810 1 -1.5134647 1 -0.9666039 1

H -3.9634169 1 -2.2060396 1 -0.8259987 1

H -2.8122644 1 1.6536160 1 -1.4120706 1

H -5.0304378 1 2.2546534 1 -1.4650765 1

H -1.9069742 1 6.4886670 1 2.3129770 1

H -1.9309246 1 6.1570960 1 0.0653015 1

H -1.6521831 1 4.0455948 1 -0.8207812 1

H -1.3317626 1 2.6037152 1 2.9608425 1

H -1.6346303 1 4.7493455 1 3.7491193 1

0

---------------------------------------- End of file **YAVSOD.mop**-----------------------------------

---------------------------------------- Begin of file **YAVSOD.arc**-----------------------------------

SUMMARY OF RM1 CALCULATION, Site No: 999

MOPAC2009 (Version: 11.03W )

Wed Aug 21 12:12:32 2013

Empirical Formula: C20 H20 N6 O10 Dy = 57 atoms

RM1 EXTERNAL=spk.inp PRECISE NOINTER XYZ BFGS T=10D GNORM=0.25 +

NOLOG GEO-OK SCFCRT=1.D-10 CHARGE=1.0

NUMERO DE COORDENAÇÃO= 8

PETERS TEST WAS SATISFIED IN BFGS OPTIMIZATION

SCF FIELD WAS ACHIEVED

HEAT OF FORMATION = 11.54660 KCAL/MOL = 48.31097 KJ/MOL

TOTAL ENERGY = -7253.19663 EV

ELECTRONIC ENERGY = -60048.82530 EV

CORE-CORE REPULSION = 52795.62867 EV

GRADIENT NORM = 0.17996

DIPOLE = 0.67935 DEBYE POINT GROUP: C1

NO. OF FILLED LEVELS = 96

CHARGE ON SYSTEM = 1

IONIZATION POTENTIAL = 12.009019 EV

HOMO LUMO ENERGIES (EV) = -12.009 -3.849

MOLECULAR WEIGHT = 666.912

COSMO AREA = 489.42 SQUARE ANGSTROMS

COSMO VOLUME = 591.99 CUBIC ANGSTROMS

MOLECULAR DIMENSIONS (Angstroms)

Atom Atom Distance

H 48 H 38 14.75392

H 48 H 38 14.61973

O 10 O 11 7.97802

SCF CALCULATIONS = 655

COMPUTATION TIME = 12 MINUTES AND 19.710 SECONDS

FINAL GEOMETRY OBTAINED CHARGE

RM1 EXTERNAL=spk.inp PRECISE NOINTER XYZ BFGS T=10D GNORM=0.25 +

NOLOG GEO-OK SCFCRT=1.D-10 CHARGE=1.0

NUMERO DE COORDENAÇÃO= 8

Dy -0.05755731 +1 0.01267527 +1 0.01366456 +1 0.0361

O 0.08567594 +1 -2.28630415 +1 -0.26930831 +1 -0.3731

O 2.26079710 +1 0.19010900 +1 -0.02494328 +1 -0.3844

O -2.30121173 +1 -0.02104667 +1 -0.57949358 +1 -0.3733

O -0.29092817 +1 2.13881865 +1 0.92553425 +1 -0.3845

O 0.31509849 +1 -0.42892406 +1 -2.39310672 +1 -0.3562

O 0.19108463 +1 1.64792248 +1 -1.82361449 +1 -0.3591

O 0.76705205 +1 -0.44212500 +1 2.30012960 +1 -0.3594

O -1.36864784 +1 -0.57639671 +1 2.02887402 +1 -0.3568

O 1.09604345 +1 1.09936915 +1 -3.71169585 +1 -0.2227

O -0.54641840 +1 -0.34845295 +1 4.01776937 +1 -0.2232

N -0.39142456 +1 -0.43492097 +1 2.83731482 +1 0.5563

N 0.57422821 +1 0.78616513 +1 -2.68484313 +1 0.5563

N -0.59589709 +1 6.05863700 +1 1.92885070 +1 -0.2533

N -6.31578270 +1 -0.24034723 +1 -1.12074703 +1 -0.2538

N 0.38361398 +1 -6.21359579 +1 -1.24080280 +1 -0.2539

N 6.27618722 +1 0.36047884 +1 0.53479532 +1 -0.2533

C 5.75243585 +1 0.60664590 +1 -0.71780267 +1 0.1164

C 4.40322558 +1 0.55635282 +1 -0.93658235 +1 -0.2968

C 3.52531313 +1 0.24442921 +1 0.14774988 +1 0.4618

C 4.09495499 +1 -0.00719572 +1 1.43683898 +1 -0.2876

C 5.44975769 +1 0.05654469 +1 1.59990023 +1 0.1079

C 0.29507254 +1 -5.84597060 +1 0.08649534 +1 0.1146

C 0.19426689 +1 -4.53015589 +1 0.44474336 +1 -0.2952

C 0.17960755 +1 -3.52337662 +1 -0.57042247 +1 0.4617

C 0.27328883 +1 -3.93185525 +1 -1.93957075 +1 -0.2882

C 0.37300790 +1 -5.26034508 +1 -2.24136774 +1 0.1083

C -5.77333943 +1 -0.55438474 +1 0.11093021 +1 0.1081

C -4.42478319 +1 -0.48756760 +1 0.31781112 +1 -0.2882

C -3.56422401 +1 -0.08718084 +1 -0.75429943 +1 0.4617

C -4.15199508 +1 0.23429584 +1 -2.01741568 +1 -0.2951

C -5.50835106 +1 0.14975176 +1 -2.16981064 +1 0.1146

C -0.36317606 +1 5.70601455 +1 0.61302239 +1 0.1079

C -0.25724513 +1 4.39333126 +1 0.24991981 +1 -0.2873

C -0.38803149 +1 3.37196593 +1 1.24449013 +1 0.4619

C -0.62890811 +1 3.76465390 +1 2.59784379 +1 -0.2970

C -0.72663541 +1 5.09357183 +1 2.90631000 +1 0.1164

H 7.28511040 +1 0.40351715 +1 0.67684613 +1 0.2787

H 6.46677451 +1 0.84294135 +1 -1.52412854 +1 0.1541

H 3.98750674 +1 0.75223583 +1 -1.93221824 +1 0.1817

H 3.42735208 +1 -0.24805407 +1 2.27703936 +1 0.1952

H 5.93252949 +1 -0.12909470 +1 2.57366155 +1 0.1545

H 0.45870398 +1 -7.20043875 +1 -1.48649582 +1 0.2785

H 0.30946510 +1 -6.65603461 +1 0.83459548 +1 0.1534

H 0.12370950 +1 -4.23444710 +1 1.49837743 +1 0.1816

H 0.26685906 +1 -3.16320041 +1 -2.72598164 +1 0.1940

H 0.44848884 +1 -5.62156211 +1 -3.28038471 +1 0.1543

H -7.32499924 +1 -0.29683127 +1 -1.25540612 +1 0.2785

H -6.47434043 +1 -0.85705413 +1 0.90637695 +1 0.1543

H -3.98086786 +1 -0.73318731 +1 1.29354063 +1 0.1945

H -3.50757979 +1 0.54410638 +1 -2.84884579 +1 0.1816

H -6.00516961 +1 0.38660189 +1 -3.12539846 +1 0.1533

H -0.67169961 +1 7.04411279 +1 2.17999958 +1 0.2787

H -0.26869088 +1 6.52625883 +1 -0.11776586 +1 0.1546

H -0.07251697 +1 4.10046728 +1 -0.79401700 +1 0.1953

H -0.73265747 +1 2.99792748 +1 3.37493355 +1 0.1816

H -0.91149482 +1 5.44231846 +1 3.93611710 +1 0.1540

---------------------------------------- End of file **YAVSOD.arc**-----------------------------------

**Holmium: QOZVOQ**

**
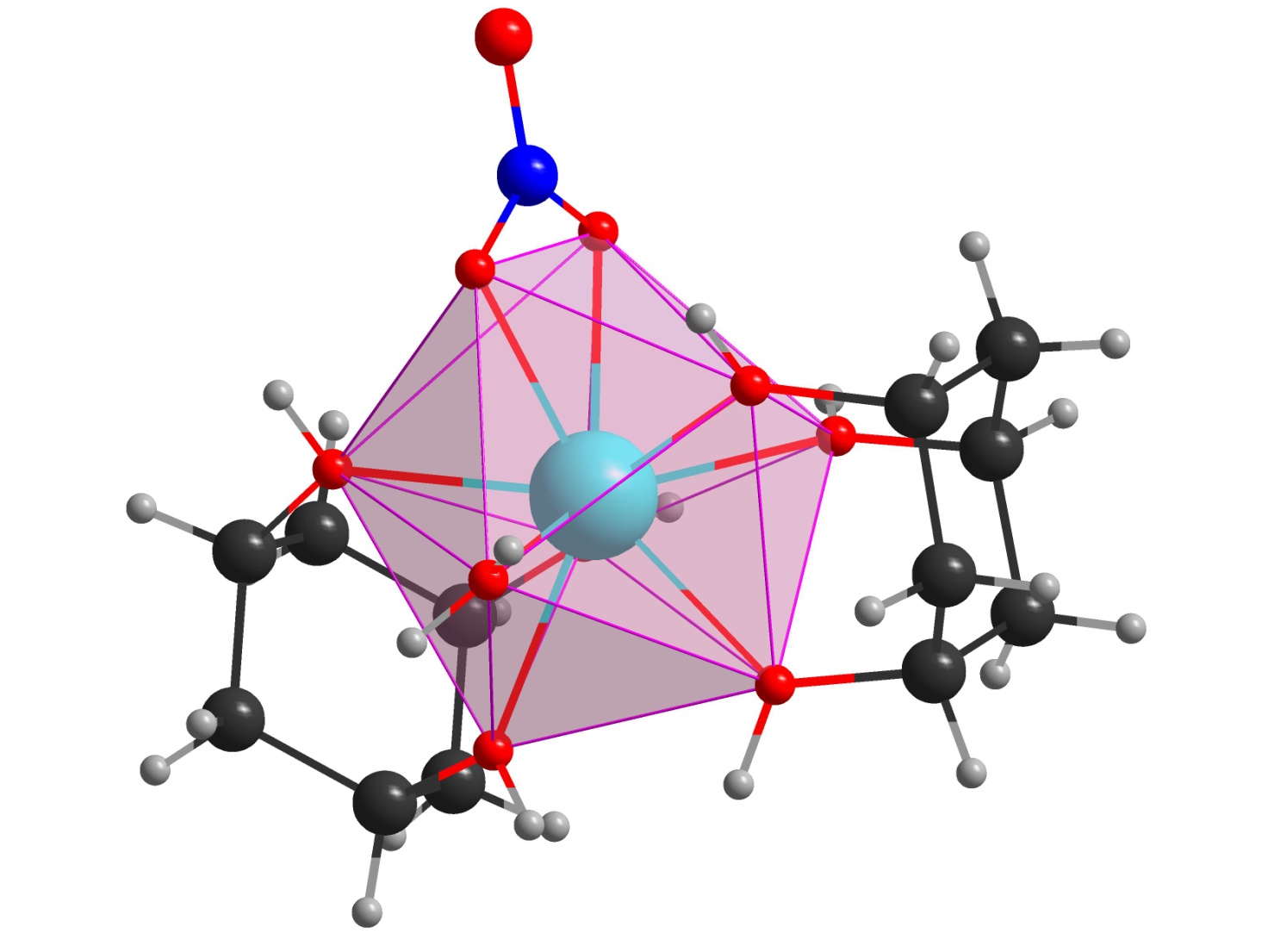
**

---------------------------------------- Begin of file **QOZVOQ.mop**-----------------------------------

RM1 EXTERNAL=spk.inp PRECISE NOINTER XYZ BFGS T=10D GNORM=0.25 +

NOLOG GEO-OK SCFCRT=1.D-10 CHARGE=2.0

NUMERO DE COORDENAÇÃO = 9

Ho 0.0000000 1 0.0000000 1 0.0000000 1

O 2.4148000 1 0.0000000 1 0.0000000 1

O 0.7146149 1 -1.7482159 1 1.3990878 1

O 0.8024369 1 -1.6976019 1 -1.4424699 1

O -1.6911833 1 1.6755359 1 0.1184374 1

O -1.0934450 1 0.0880158 1 -2.1692056 1

O 0.8339253 1 1.8764607 1 -1.2026786 1

O -0.9169680 1 0.2164949 1 2.2617931 1

O 0.8841461 1 1.3338302 1 1.8599312 1

O -1.8452969 1 -1.4954372 1 0.0878920 1

O 0.0606163 1 1.3124572 1 3.8832500 1

H 2.8019495 1 0.8449258 1 0.0000000 1

H 0.2151153 1 -1.8499817 1 2.1764551 1

H 0.3505889 1 -1.7653259 1 -2.2534172 1

H -2.1677062 1 1.6978447 1 0.9169988 1

H -1.2037675 1 -0.7332704 1 -2.5942204 1

H 1.7523495 1 2.0079405 1 -1.1330061 1

H -1.9542233 1 -2.1369513 1 0.5850759 1

H -2.4730434 1 -1.1814501 1 -0.4524673 1

N 0.0325715 1 0.9610509 1 2.6973364 1

C 3.3674839 1 -1.1099026 1 -0.0009705 1

H 4.2771626 1 -0.7440727 1 0.0141753 1

C 3.1610192 1 -1.9500874 1 1.2434190 1

H 3.1903850 1 -1.3699045 1 2.0208480 1

H 3.8934591 1 -2.5816824 1 1.3202796 1

C 1.8348870 1 -2.7228781 1 1.2518292 1

H 1.8236967 1 -3.3531046 1 2.0023525 1

C 1.6210165 1 -3.4765804 1 -0.0552529 1

H 2.2047315 1 -4.2512456 1 -0.0715210 1

H 0.7059865 1 -3.7972944 1 -0.0861840 1

C 1.8828767 1 -2.6505653 1 -1.2686832 1

H 1.8864455 1 -3.2473895 1 -2.0442128 1

C 3.1808906 1 -1.8824206 1 -1.2698309 1

H 3.9193633 1 -2.5010369 1 -1.3832853 1

H 3.1884289 1 -1.2683440 1 -2.0227318 1

C -2.0962633 1 2.6737357 1 -0.8677510 1

H -2.7968459 1 3.2339667 1 -0.4728822 1

C -2.6878334 1 1.9395715 1 -2.0819746 1

H -3.3320335 1 1.2812417 1 -1.7762323 1

H -3.1545487 1 2.5757641 1 -2.6454992 1

C -1.6007867 1 1.2408085 1 -2.9006873 1

H -2.0027538 1 0.9230664 1 -3.7370073 1

C -0.4501314 1 2.1556053 1 -3.2451219 1

H 0.2501079 1 1.6348460 1 -3.6681902 1

H -0.7548359 1 2.8172641 1 -3.8857910 1

C 0.1289308 1 2.8706784 1 -2.0419175 1

H 0.7780147 1 3.5365795 1 -2.3534274 1

C -0.9216185 1 3.5623015 1 -1.2397512 1

H -1.2508306 1 4.3220307 1 -1.7440937 1

H -0.5187408 1 3.9042665 1 -0.4250214 1

---------------------------------------- End of file **QOZVOQ.mop**-----------------------------------

---------------------------------------- Begin of file **QOZVOQ.arc**-----------------------------------

SUMMARY OF RM1 CALCULATION, Site No: 999

MOPAC2009 (Version: 11.03W )

Fri Aug 16 15:21:22 2013

Empirical Formula: C12 H26 N O10 Ho = 50 atoms

RM1 EXTERNAL=spk.inp PRECISE NOINTER XYZ BFGS T=10D GNORM=0.25 +

NOLOG GEO-OK SCFCRT=1.D-10 CHARGE=2.0

NUMERO DE COORDENAÇÃO = 9

PETERS TEST WAS SATISFIED IN BFGS OPTIMIZATION

SCF FIELD WAS ACHIEVED

HEAT OF FORMATION = 113.78508 KCAL/MOL = 476.07679 KJ/MOL

TOTAL ENERGY = -5283.87341 EV

ELECTRONIC ENERGY = -41299.00349 EV

CORE-CORE REPULSION = 36015.13007 EV

GRADIENT NORM = 0.20707

DIPOLE = 8.72281 DEBYE POINT GROUP: C1

NO. OF FILLED LEVELS = 70

CHARGE ON SYSTEM = 2

IONIZATION POTENTIAL = 18.543275 EV

HOMO LUMO ENERGIES (EV) = -18.543 -9.330

MOLECULAR WEIGHT = 509.268

COSMO AREA = 313.64 SQUARE ANGSTROMS

COSMO VOLUME = 412.70 CUBIC ANGSTROMS

MOLECULAR DIMENSIONS (Angstroms)

Atom Atom Distance

H 29 H 49 9.69197

H 42 O 11 7.88830

H 19 H 22 6.58514

SCF CALCULATIONS = 266

COMPUTATION TIME = 3 MINUTES AND 38.823 SECONDS

FINAL GEOMETRY OBTAINED CHARGE

RM1 EXTERNAL=spk.inp PRECISE NOINTER XYZ BFGS T=10D GNORM=0.25 +

NOLOG GEO-OK SCFCRT=1.D-10 CHARGE=2.0

NUMERO DE COORDENAÇÃO = 9

Ho 0.04804756 +1 0.03705134 +1 -0.00605007 +1 0.1982

O 2.44902852 +1 -0.09414408 +1 0.24804243 +1 -0.3432

O 0.65811392 +1 -1.88834058 +1 1.29874318 +1 -0.3492

O 1.00038881 +1 -1.54116413 +1 -1.53362363 +1 -0.3422

O -1.73381490 +1 1.67545385 +1 0.03975450 +1 -0.3475

O -1.18031786 +1 0.09502743 +1 -2.07857476 +1 -0.3518

O 0.76126677 +1 2.02098254 +1 -1.12051437 +1 -0.3391

O -0.63549807 +1 0.36522723 +1 2.32261042 +1 -0.4337

O 0.91736829 +1 1.64917873 +1 1.63731202 +1 -0.4214

O -1.86828315 +1 -1.31719877 +1 0.43452245 +1 -0.3480

O 0.25452874 +1 1.79602322 +1 3.71165667 +1 -0.0279

H 2.91011484 +1 0.68934828 +1 0.60420377 +1 0.3083

H 0.31972104 +1 -1.94601987 +1 2.21417546 +1 0.3133

H 0.88678853 +1 -1.38568852 +1 -2.48806186 +1 0.2973

H -2.16529318 +1 1.85736333 +1 0.89438247 +1 0.3056

H -1.71393599 +1 -0.68690603 +1 -2.30654061 +1 0.2946

H 1.53566107 +1 2.49623094 +1 -0.76236829 +1 0.3107

H -1.92104782 +1 -2.28268346 +1 0.48597379 +1 0.3176

H -2.67721778 +1 -0.98489518 +1 0.85771002 +1 0.3244

N 0.18763899 +1 1.31126003 +1 2.64775186 +1 0.5471

C 3.40318014 +1 -1.17035880 +1 0.24185984 +1 0.1565

H 4.42963984 +1 -0.72999262 +1 0.40858469 +1 0.1317

C 3.05035785 +1 -2.12971667 +1 1.37603109 +1 -0.2419

H 3.06923440 +1 -1.60989955 +1 2.36264597 +1 0.1384

H 3.86272179 +1 -2.88983696 +1 1.47264272 +1 0.1580

C 1.72061456 +1 -2.84636300 +1 1.15738034 +1 0.1517

H 1.57430159 +1 -3.64529857 +1 1.94301065 +1 0.1319

C 1.58634300 +1 -3.46416710 +1 -0.23281039 +1 -0.2159

H 2.21989525 +1 -4.38510430 +1 -0.26699812 +1 0.1596

H 0.55523439 +1 -3.85594667 +1 -0.39935175 +1 0.1274

C 2.01608913 +1 -2.55254447 +1 -1.37817631 +1 0.1536

H 2.06155284 +1 -3.14623793 +1 -2.33824962 +1 0.1288

C 3.34254048 +1 -1.84259812 +1 -1.12645881 +1 -0.2329

H 4.17381678 +1 -2.58516743 +1 -1.19929127 +1 0.1587

H 3.57367776 +1 -1.11722511 +1 -1.94085475 +1 0.1324

C -2.20357333 +1 2.66181420 +1 -0.89570726 +1 0.1579

H -3.03842178 +1 3.24175236 +1 -0.40379848 +1 0.1321

C -2.73310778 +1 1.92541461 +1 -2.12341622 +1 -0.2363

H -3.56510648 +1 1.23564760 +1 -1.85162173 +1 0.1300

H -3.22438066 +1 2.66402375 +1 -2.80234080 +1 0.1595

C -1.63576587 +1 1.19471808 +1 -2.89055212 +1 0.1543

H -2.04361513 +1 0.76613374 +1 -3.85316440 +1 0.1290

C -0.42207788 +1 2.06949176 +1 -3.19268000 +1 -0.2165

H 0.40962123 +1 1.46586154 +1 -3.62787463 +1 0.1299

H -0.69725725 +1 2.76434737 +1 -4.02488116 +1 0.1602

C 0.07201438 +1 2.91060950 +1 -2.01808645 +1 0.1577

H 0.81846481 +1 3.67500246 +1 -2.38674506 +1 0.1327

C -1.04650943 +1 3.59704734 +1 -1.23828728 +1 -0.2423

H -1.44052297 +1 4.44963282 +1 -1.84273535 +1 0.1583

H -0.64907715 +1 4.09590505 +1 -0.32247217 +1 0.1426

---------------------------------------- End of file **QOZVOQ.arc**-----------------------------------

**Erbium: KOZBUW**

**
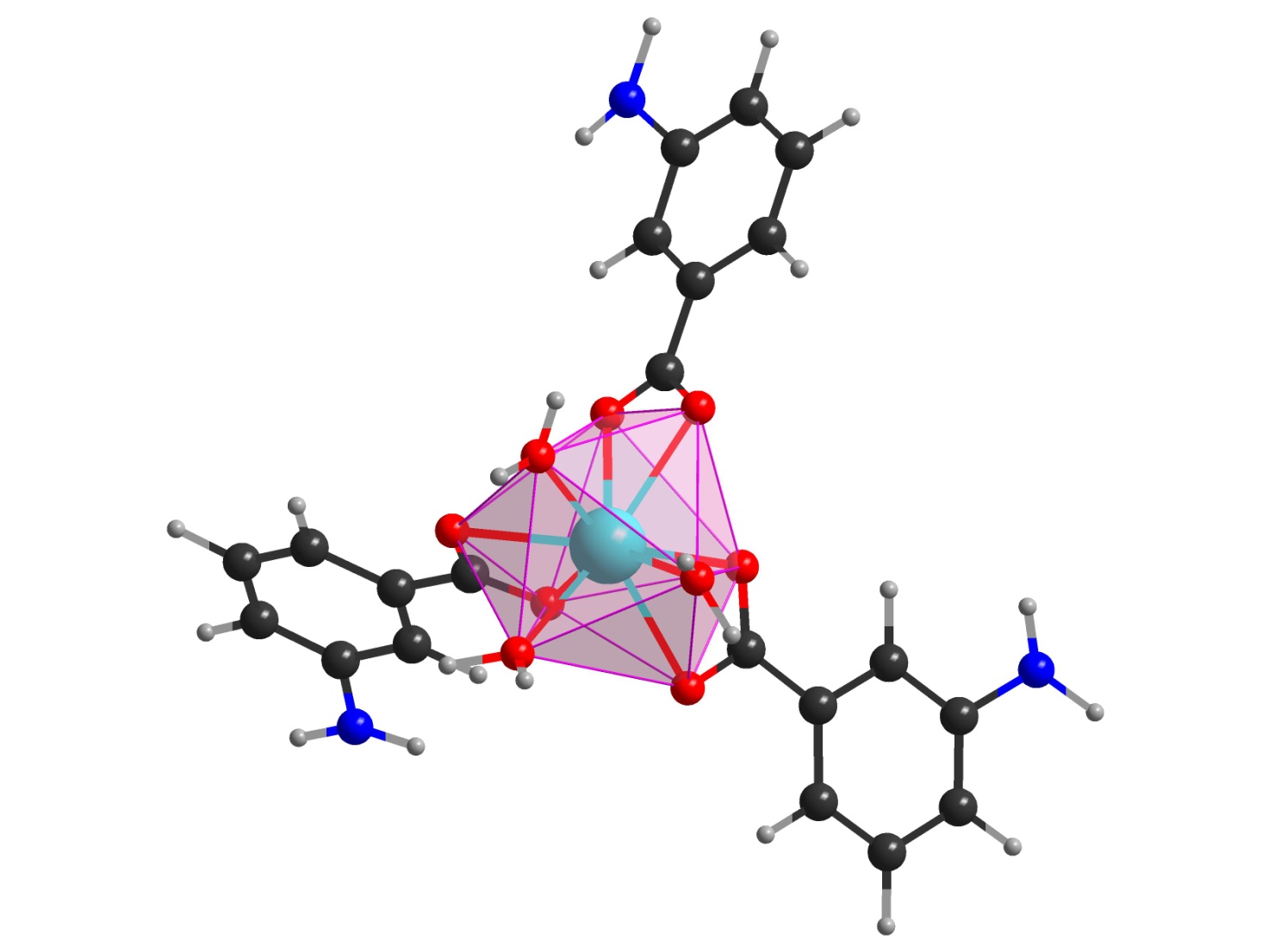
**

---------------------------------------- Begin of file **KOZBUW.mop**-----------------------------------

RM1 EXTERNAL=spk.inp PRECISE NOINTER XYZ BFGS T=10D GNORM=0.25 +

NOLOG GEO-OK SCFCRT=1.D-10

NUMERO DE COORDENAÇÃO= 9

Er 0.0000000 1 0.0000000 1 0.0000000 1

O 0.5579482 1 0.4065506 1 2.3107807 1

O 0.5580178 1 -2.1932522 1 0.8334012 1

O -1.4346214 1 -0.3628535 1 1.9336272 1

O 0.6663721 1 -1.9359535 1 -1.3174554 1

O -1.5385786 1 1.8054204 1 0.1026330 1

O -1.9623691 1 -0.9192660 1 -0.9702360 1

O 2.4116000 1 0.0000000 1 0.0000000 1

O 1.4595238 1 1.9486519 1 0.0000000 1

O 0.0466475 1 0.8633530 1 -2.2111697 1

N 7.3870414 1 0.9767299 1 -0.4897113 1

C 2.5151169 1 1.2251829 1 0.0112801 1

C 3.8099431 1 1.9026816 1 0.0007745 1

C 4.9813283 1 1.1269920 1 -0.1362546 1

C 6.2288270 1 1.7711721 1 -0.1736615 1

C 6.3249480 1 3.1023588 1 -0.0343844 1

C 5.1684581 1 3.9042007 1 0.1313555 1

C 3.9296234 1 3.3092348 1 0.1291756 1

C -0.5288579 1 0.0306107 1 2.7477172 1

C 0.7991820 1 -2.6676537 1 -0.2755096 1

H 4.9156877 1 0.0494462 1 -0.2105971 1

H 7.3002641 1 3.5723719 1 -0.0517638 1

H 5.2583433 1 4.9825366 1 0.2608791 1

H 3.0452291 1 3.9041620 1 0.2207203 1

H 7.2193424 1 0.0477454 1 -0.4888354 1

H 8.4838867 1 1.4630610 1 -0.4414090 1

H 0.3381585 1 1.7573758 1 -2.6905701 1

H -0.7078655 1 0.8480819 1 -2.9784267 1

H -2.4501072 1 2.0233242 1 0.5887886 1

H -1.9640871 1 2.3998555 1 -0.6873107 1

H -2.2038706 1 -1.5959160 1 -1.7441597 1

H -2.8749926 1 -0.4385297 1 -1.2782347 1

C -0.8480436 1 0.0472656 1 4.1725074 1

C 1.2024859 1 -4.0590964 1 -0.4614817 1

C 0.0804199 1 0.6147002 1 5.0712517 1

C -2.0560770 1 -0.4923027 1 4.6845922 1

C 1.2120823 1 -4.9191963 1 0.6568988 1

C 1.5912090 1 -4.5717593 1 -1.7255741 1

C -0.2297016 1 0.6592821 1 6.4406676 1

H 1.0195142 1 1.0100563 1 4.7066255 1

C -2.3088195 1 -0.4705864 1 6.0344633 1

H -2.7700606 1 -0.9126683 1 4.0079991 1

C 1.5734669 1 -6.2657735 1 0.4824367 1

H 0.9439839 1 -4.5436136 1 1.6354531 1

C 1.9794530 1 -5.8831321 1 -1.8523426 1

H 1.5739616 1 -3.9291213 1 -2.5797953 1

N 0.6523938 1 1.3982533 1 7.3056980 1

C -1.3700430 1 0.1285900 1 6.9091631 1

H -3.2246036 1 -0.9141112 1 6.4274899 1

N 1.4044110 1 -7.1569350 1 1.6004650 1

C 1.9540470 1 -6.7304843 1 -0.7179682 1

H 2.3066057 1 -6.2667318 1 -2.8197542 1

H 1.4575473 1 1.6589820 1 6.8911305 1

H 0.4801024 1 1.3856586 1 8.4944109 1

H -1.5775169 1 0.1626526 1 7.9710485 1

H 1.2098406 1 -6.7151629 1 2.4104281 1

H 1.7863602 1 -8.2935280 1 1.5286841 1

H 2.2415495 1 -7.7687202 1 -0.8207559 1

0

---------------------------------------- End of file **KOZBUW.mop**-----------------------------------

---------------------------------------- Begin of file **KOZBUW.arc**-----------------------------------

SUMMARY OF RM1 CALCULATION, Site No: 999

MOPAC2009 (Version: 11.03W )

Fri Aug 16 14:10:33 2013

Empirical Formula: C21 H24 N3 O9 Er = 58 atoms

RM1 EXTERNAL=spk.inp PRECISE NOINTER XYZ BFGS T=10D GNORM=0.25 +

NOLOG GEO-OK SCFCRT=1.D-10

NUMERO DE COORDENAÇÃO= 9

PETERS TEST WAS SATISFIED IN BFGS OPTIMIZATION

SCF FIELD WAS ACHIEVED

HEAT OF FORMATION = -338.16357 KCAL/MOL = -1414.87639 KJ/MOL

TOTAL ENERGY = -6508.06722 EV

ELECTRONIC ENERGY = -53866.32333 EV

CORE-CORE REPULSION = 47358.25611 EV

GRADIENT NORM = 0.24025

DIPOLE = 5.36047 DEBYE POINT GROUP: C1

NO. OF FILLED LEVELS = 90

IONIZATION POTENTIAL = 8.595483 EV

HOMO LUMO ENERGIES (EV) = -8.595 -3.073

MOLECULAR WEIGHT = 629.695

COSMO AREA = 463.14 SQUARE ANGSTROMS

COSMO VOLUME = 557.62 CUBIC ANGSTROMS

MOLECULAR DIMENSIONS (Angstroms)

Atom Atom Distance

H 22 H 49 11.46174

H 52 H 54 11.20285

H 57 H 30 10.76061

SCF CALCULATIONS = 471

COMPUTATION TIME = 15 MINUTES AND 4.057 SECONDS

FINAL GEOMETRY OBTAINED CHARGE

RM1 EXTERNAL=spk.inp PRECISE NOINTER XYZ BFGS T=10D GNORM=0.25 +

NOLOG GEO-OK SCFCRT=1.D-10

NUMERO DE COORDENAÇÃO= 9

Er -0.34232338 +1 0.25924324 +1 -0.37774472 +1 -0.8835

O 0.35634880 +1 1.06580743 +1 1.78377297 +1 -0.3374

O -0.20114564 +1 -1.85404713 +1 0.77530087 +1 -0.3361

O -1.76093826 +1 0.62284832 +1 1.53103468 +1 -0.3643

O -0.28404804 +1 -1.91604969 +1 -1.40037649 +1 -0.3658

O -1.47523426 +1 2.33823147 +1 -0.68234922 +1 -0.2247

O -2.56165802 +1 -0.32962965 +1 -1.03197005 +1 -0.2247

O 2.02799453 +1 -0.13858994 +1 -0.57193868 +1 -0.3364

O 1.33085641 +1 1.88872623 +1 -0.95449434 +1 -0.3643

O -0.40289426 +1 0.59566826 +1 -2.74102854 +1 -0.2244

N 6.63389245 +1 0.44651462 +1 1.61376815 +1 -0.4505

C 2.24370627 +1 1.13477724 +1 -0.39783268 +1 0.5054

C 3.47384134 +1 1.67189283 +1 0.19799410 +1 -0.0583

C 4.45606426 +1 0.78829981 +1 0.63250925 +1 -0.1531

C 5.64480761 +1 1.30486257 +1 1.17638880 +1 0.1821

C 5.82158832 +1 2.69772298 +1 1.27581800 +1 -0.1850

C 4.82357576 +1 3.55011515 +1 0.83643421 +1 -0.0516

C 3.64394327 +1 3.05118558 +1 0.29432788 +1 -0.1024

C -0.71495152 +1 0.54518302 +1 2.31277683 +1 0.5057

C 0.25551583 +1 -2.40544856 +1 -0.31341417 +1 0.5059

H 4.30220119 +1 -0.29323489 +1 0.54924467 +1 0.1412

H 6.74101182 +1 3.11512690 +1 1.69720315 +1 0.1185

H 4.96433583 +1 4.63315693 +1 0.91587029 +1 0.1104

H 2.86526198 +1 3.73880745 +1 -0.04876385 +1 0.1290

H 6.52670268 +1 -0.54452995 +1 1.53615411 +1 0.2318

H 7.49222774 +1 0.79817596 +1 1.98602008 +1 0.2294

H 0.44966657 +1 0.72671486 +1 -3.17813451 +1 0.2883

H -0.85975230 +1 -0.10961422 +1 -3.22135264 +1 0.2849

H -1.61881385 +1 2.87511470 +1 0.10882163 +1 0.2881

H -1.05780341 +1 2.91245531 +1 -1.34044385 +1 0.2854

H -2.80940934 +1 -1.25969318 +1 -0.93930993 +1 0.2883

H -3.24778190 +1 0.18985185 +1 -0.58902534 +1 0.2851

C -0.78702434 +1 0.09134541 +1 3.70755486 +1 -0.0587

C 1.13981337 +1 -3.57781707 +1 -0.31664517 +1 -0.0587

C 0.33393947 +1 0.22685317 +1 4.51960858 +1 -0.1538

C -1.97817704 +1 -0.43976040 +1 4.19680073 +1 -0.1018

C 1.54999235 +1 -4.11861941 +1 0.89719503 +1 -0.1531

C 1.52807842 +1 -4.14058975 +1 -1.53042744 +1 -0.1025

C 0.25494547 +1 -0.17845067 +1 5.86311662 +1 0.1822

H 1.26218199 +1 0.64518132 +1 4.11506596 +1 0.1412

C -2.04268904 +1 -0.84071438 +1 5.52708374 +1 -0.0516

H -2.85431914 +1 -0.54410476 +1 3.54998905 +1 0.1290

C 2.37002866 +1 -5.26023973 +1 0.89195861 +1 0.1821

H 1.23521218 +1 -3.65948992 +1 1.84079831 +1 0.1412

C 2.34079095 +1 -5.26917198 +1 -1.52245909 +1 -0.0516

H 1.20287780 +1 -3.70691683 +1 -2.48073288 +1 0.1289

N 1.35470423 +1 -0.05082456 +1 6.68805215 +1 -0.4505

C -0.94631531 +1 -0.71582718 +1 6.36254403 +1 -0.1849

H -2.97525158 +1 -1.26018544 +1 5.91885677 +1 0.1104

N 2.78923137 +1 -5.81429875 +1 2.08509206 +1 -0.4505

C 2.76334783 +1 -5.83403519 +1 -0.33164940 +1 -0.1848

H 2.64966109 +1 -5.71728403 +1 -2.47268029 +1 0.1104

H 2.20961230 +1 0.34161114 +1 6.34907828 +1 0.2318

H 1.31086636 +1 -0.32125846 +1 7.64920640 +1 0.2294

H -1.02330884 +1 -1.03611503 +1 7.40593344 +1 0.1186

H 2.50616673 +1 -5.42445806 +1 2.96126344 +1 0.2318

H 3.35791927 +1 -6.63608770 +1 2.09872679 +1 0.2294

H 3.40060424 +1 -6.72320987 +1 -0.35205866 +1 0.1186

---------------------------------------- End of file **KOZBUW.arc**-----------------------------------

1. **References** ([back to contents](#Contents))
2. Allen F.H. (2002) Acta Crystallogr. B 58: 380-388.
3. Bruno I.J., Cole J.C., Edgington P.R., Kessler M., Macrae C.F., McCabe P., Pearson J., Taylor R. (2002) Acta Crystallogr. B 58: 389-397.
4. Allen F.H., Motherwell W.D.S. (2002) Acta Crystallogr. B 58: 407-422.
